# Lupus and Cutaneous Tuberculosis

**Published:** 1888-09

**Authors:** 


### Lupus and Cutaneous Tuberculosis.

At the last meeting of the German Scientific and Medical Association, Professor Doutrelepont read a paper on this subject, in which he said that genuine cutaneous tuberculosis has been observed but very seldom, and so far as he knows never in a case of lupus. But he had recently observed and treated two such cases. The first case was that of a woman, 36 years old, who had suffered from "glands" since she was 20 years old. Two years ago a nodule formed on the upper lip, immediately under the septum mobile, and in a short time several similar efflorescences covered both cheeks. During this time the first nodule grew into a large ulcer, and later, small ulcers appeared on the mucous membrane of both lips, nodules on the gums, and a large ulcer in the middle of the tongue. The patient had a cough that was periodical at first, but afterwards became constant. She was small, delicate, emaciated and anæmic. At the center of the upper lip was a rather deep ulcer, about the size of a quarter dollar, and its edges were undermined, and the pale, reddish center of which was covered with small, gray, nodule-like protuberances. There was a small amount of secretion, of a sero-purulent nature. The edges and surroundings of the ulcer did not seem to be much inflamed, but they were very painful to touch. On the cheeks were numerous brownish red nodules, singly or in groups as large as a bean; they were soft and easily pressed in, and between them cicatricial tissue was seen. Two flat ulcers, with serrated edges, as large a bean, were on the mucous membrane of the lower lip; their bottoms were almost flat, and on them could be seen gray miliary prominences. There was a similar ulcer on the upper lip. The gums were also affected, and on the tongue was a deep, crater-like ulcer. All the ulcers were painful to touch, and interfered with the patient's eating. Examination of the lungs showed dullness high up to the second rib on the left, where bronchial breathing and a slightly metallic rhoncus was heard. In the granulations of the ulcers and in the sputa many tubercle bacilli were found. The lupus on the face and the lingual ulcer were treated with a 10

per cent. pyrogallic acid salve, and sublimate bandanges 1 :1000. Galvanic cauterization was used several times a day on the ulcers of the mucous membrane of the lips, the gum nodules and the lingual ulcer, and they were painted twice a day with a 1 per cent. sublimate solution. This treatment had a good effect on the lesions of the skin and mucous membrane, but the patient grew weaker and the pulmonary affection spread rapidly; diarrhœa set in, and the pressure on the ileo-cæcal region became very painful. She was discharged from the hospital at the desire of her family. At that time the lupus of the cheek was cicatrized, the ulcer on the lip was filled with good granulations, the ulcers on the mucous membrane were healed, and the large ulcer on the tongue was cicatrized, but meanwhile cavernous formation had begun on the left of the tip of the tongue.

In the second case tuberculosis of the subcutaneous connective tissue also existed to some degree, and distinctly on the chin; the patient had advanced tuberculosis of the lungs, and the sputum was full of tubercle bacilli. This form of skin-tuberculosis is almost always secondary, and seen in cases of advanced lung-tuberculosis around the mouth or the anus. Slight abrasions of the mucous membrane afford entrance to the bacilli, which produce the miliary nodules that eventually ulcerate.—*Deutche medicinishe Wochenschrift*, No. 43, 1887.

